# Life-Cycle-Dependent Toxicities of Mono- and Bifunctional Alkylating Agents in the 3R-Compliant Model Organism *C. elegans*

**DOI:** 10.3390/cells12232728

**Published:** 2023-11-29

**Authors:** Joanna Ruszkiewicz, Lisa Endig, Ebru Güver, Alexander Bürkle, Aswin Mangerich

**Affiliations:** 1 Molecular Toxicology Group, Department of Biology, University of Konstanz, 78457 Konstanz, Germanyalexander.buerkle@uni-konstanz.de (A.B.); 2 Nutritional Toxicology, Institute Nutritional Science, University of Potsdam, 14469 Potsdam, Germany

**Keywords:** *C. elegans*, alkylating agents, mustards, life cycle toxicities, neurotoxicity, NAD^+^

## Abstract

*Caenorhabditis elegans (C. elegans)* is gaining recognition and importance as an organismic model for toxicity testing in line with the 3Rs principle (replace, reduce, refine). In this study, we explored the use of *C. elegans* to examine the toxicities of alkylating sulphur mustard analogues, specifically the monofunctional agent 2-chloroethyl-ethyl sulphide (CEES) and the bifunctional, crosslinking agent mechlorethamine (HN2). We exposed wild-type worms at different life cycle stages (from larvae L1 to adulthood day 10) to CEES or HN2 and scored their viability 24 h later. The susceptibility of *C. elegans* to CEES and HN2 paralleled that of human cells, with HN2 exhibiting higher toxicity than CEES, reflected in LC_50_ values in the high µM to low mM range. Importantly, the effects were dependent on the worms’ developmental stage as well as organismic age: the highest susceptibility was observed in L1, whereas the lowest was observed in L4 worms. In adult worms, susceptibility to alkylating agents increased with advanced age, especially to HN2. To examine reproductive effects, L4 worms were exposed to CEES and HN2, and both the offspring and the percentage of unhatched eggs were assessed. Moreover, germline apoptosis was assessed by using *ced-1p*::GFP (MD701) worms. In contrast to concentrations that elicited low toxicities to L4 worms, CEES and HN2 were highly toxic to germline cells, manifesting as increased germline apoptosis as well as reduced offspring number and percentage of eggs hatched. Again, HN2 exhibited stronger effects than CEES. Compound specificity was also evident in toxicities to dopaminergic neurons–HN2 exposure affected expression of dopamine transporter DAT-1 (strain BY200) at lower concentrations than CEES, suggesting a higher neurotoxic effect. Mechanistically, nicotinamide adenine dinucleotide (NAD^+^) has been linked to mustard agent toxicities. Therefore, the NAD^+^-dependent system was investigated in the response to CEES and HN2 treatment. Overall NAD^+^ levels in worm extracts were revealed to be largely resistant to mustard exposure except for high concentrations, which lowered the NAD^+^ levels in L4 worms 24 h post-treatment. Interestingly, however, mutant worms lacking components of NAD^+^-dependent pathways involved in genome maintenance, namely *pme-2*, *parg-2*, and *sirt-2.1* showed a higher and compound-specific susceptibility, indicating an active role of NAD^+^ in genotoxic stress response. In conclusion, the present results demonstrate that *C. elegans* represents an attractive model to study the toxicology of alkylating agents, which supports its use in mechanistic as well as intervention studies with major strength in the possibility to analyze toxicities at different life cycle stages.

## 1. Introduction

*Caenorhabditis elegans* (*C. elegans*) is one of the most popular models in developmental biology and aging research [1,2]. Furthermore, worms are emerging as an attractive organismic model in toxicity testing in compliance with the 3Rs (replace, reduce, refine) concept to avoid the use of vertebrate animal testing [3,4,5,6,7]. Here, we investigated the potential use of the nematode to study toxicities of alkylating sulphur mustard (SM; bis[2-chloroethyl)]sulphide) analogues. SM is a chemical warfare agent which, despite being banned by the Chemical Weapons Convention (CWC), is still occasionally released in military conflicts or can be released from old depots [8]. SM skin exposure leads to blister formation, and upon absorption of SM can cause multiple diseases, such as pulmonary, cardiovascular, gastrointestinal, or immunological pathologies or cancer [9]. To date, no effective causal treatment against SM-induced pathology exists [10]. Notably, SM-derivatives, i.e., nitrogen mustards, such as chlorambucil or melphalan, demonstrate cytostatic properties that are exploited in chemotherapy [11,12]. Thus, deciphering toxicities of mustards is of high relevance on multiple levels—i.e., in the search for SM antidotes on the one hand and safer and more efficient anti-cancer therapy on the other hand.

SM is a bi-functional alkylating agent able to alkylate various macromolecules and impair cellular functions. The alkylation of DNA (particularly guanine nucleotides) is characterized by the formation of mono- and bi-adducts promoting the formation of intra-strand crosslinks and also of inter-strand crosslinks (ICLs) and is considered a key event contributing to SM toxicity [13,14]. In basic research, due to legal restrictions, SM is replaced with surrogate substances, such as the mono-functional alkylating agent 2-chloroethyl ethyl sulfide (CEES, aka half mustard), and the bifunctional crosslinking agent mechlorethamine (HN2). CEES and HN2 have different DNA alkylation profiles: HN2, similarly to SM, induces mostly monoadducts at guanines but also ICLs [15], whereas CEES solely induces monoadducts, which in the course of DNA repair can be converted into DNA strand breaks (SBs) [16,17,18]. These chemicals were investigated in the present study in *C. elegans* for core toxicity endpoints such as survival at different life cycle stages, fertility, germline apoptosis, and neurotoxicity.

Additionally, the role of nicotinamide adenine dinucleotide (NAD^+^), which is a versatile cellular factor in energy metabolism, redox regulation, and genome maintenance [19,20], was examined. Previous studies had shown that mustard exposure resulted in a partial depletion of NAD^+^ levels in various experimental conditions [21,22,23,24,25,26]. This NAD^+^ reduction has been attributed to the overactivation of NAD^+^-consuming enzymes, such as diphtheria-toxin-like ADP ribosyl transferases (ARTDs, aka PARPs) in the response to DNA damage [27,28]. ARTDs utilise NAD^+^ to synthesise poly(ADP-ribose) (PAR), which serves as a signalling and scaffold molecule to recruit repair proteins to the site of the DNA damage [20,29]. A single ARTD molecule can consume hundreds of NAD^+^ molecules during a short time period. Thus, a substantial deficit of this cofactor can be created in the event of massive DNA damage [29]. PAR is mostly degraded by poly(ADP-ribose) glycohydrolases (PARGs), which cleave the O-glycosidic ribose–ribose bonds that link the ADP-ribose monomers within PAR. PARG is crucial for the dynamic regulation of PAR levels in cells, and its deficiency results in disruption of DNA repair and apoptosis and higher sensitivity to genotoxic stress [30]. Importantly, PAR degradation does not restore NAD^+^ levels; instead, NAD^+^ needs to be resynthesized, e.g., from nicotinamide (NAM) which is a by-product of ARTDs’ catalytic activity [29]. The mustard-induced ARTDs overactivation and subsequent reduction of the cellular NAD^+^ pool can affect the activity level of multiple NAD^+^-dependent pathways, including sirtuins (SIRTs) activity, energy metabolism, or redox homeostasis, leading to pathological conditions and cell death, as reviewed elsewhere [20,28]. As NAD^+^ synthesis and metabolism are highly conserved [31,32], these pathways can be conveniently studied in the nematode.

## 2. Materials and Methods

### 2.1. C. elegans Maintenance

The following *C. elegans* strains were used: wild type (WT) N2 Bristol, RB1042 [*pme-1(ok988) I*], VC1171 [*pme-2(ok344) II* ], VC130 [*parg-1(gk120) IV*], VC641 [*parg-2(ok980) IV*], VC199 [*sirt-2.1(ok434) IV*], RB654 [*sirt-2.3(ok444) X*], MD701 [bcIs39((*lim-7*)*ced-1p*::GFP+*lin-15*(+)) *V*], BY200 [*vtIs1*(*dat-1p*::GFP) *V*]. The sources of all strains were the Caenorhabditis Genetic Center (CGC; University of Minnesota), except for BY200 (original source: Blakely laboratory, Vanderbilt University Medical Center). Strains were grown on nematode growth medium (NGM) seeded with *E. coli* strain OP50 and maintained at 20 °C. Synchronous populations were obtained by isolating embryos from gravid worms with a bleaching solution (1% NaOCl and 0.25 M NaOH), followed by separating embryos from debris by sucrose gradient centrifugation. After the procedure, embryos were washed three times with M9 buffer (22 mM KH_2_PO_4_, 42 mM Na_2_HPO_4_, 86 mM NaCl, 1 mM MgSO_4_). Such embryos were incubated further for 18 h in M9 to obtain a population of developmentally arrested larvae L1. To obtain the L4 stage, L1s were transferred onto NGM plates and allowed to grow for 48 h at 20 °C. To study adult worms, 50 µM 5’-fluorodeoxyuridine (FUdR) (Sigma–Aldrich, St. Louis, MO, USA) was added to NGM to block nematode reproduction [33]. Synchronous L4s were moved onto NGM + FUdR plates and allowed to grow for a day (A1), five days (A5), or ten days (A10). Of note, A5 worms were transferred on fresh NGM + FUdR and cultivated until A10. For survival experiments with mutant strains A1 worms were cultivated on regular NGM plates.

### 2.2. Treatment with Mustards

Premixes (100×) of CEES or HN2 (both Sigma–Aldrich, St. Louis, MO, USA) solutions were freshly prepared in solvent containing 95% EtOH and 0.5% HCl (*v*/*v*) directly before treatments. Due to the high toxicity and carcinogenicity, these chemicals were handled with extreme caution in the proper laboratory settings. Synchronized worms at different life cycle stages were exposed to CEES or HN2 in M9 for 30 min at room temperature (RT) without a food source. Additionally, negative control samples, i.e., 1% solvent (0 mM), as well as M9 only were prepared. Of note is that the addition of the chemicals did not alter the pH of M9. After toxic treatment, worms were washed twice with M9 by centrifugation (1000× *g*, 1 min, RT).

### 2.3. Survival and Reproduction

For survival assays, after treatments of synchronized worms at different life cycle stages, approximately 30 worms were placed on 35 mm NGM plates in triplicate and incubated at 20 °C, and the viability of the worms was scored 24 h later. Worm survival was determined as a percentage of the control value (0 mM), and a non-linear regression model (sigmoidal, 4PL, X is log[concentration]) was applied to determine a lethal concentration of 50% (LC_50_) using GraphPad Prism v.9.2.0 software (GraphPad Software, Boston, MA, USA).

For reproduction assays, worms were exposed to CEES or HN2 for 30 min during the L4 stage. Next, worms were placed on NGM plates and incubated at 20 °C for 24 h. Adult hermaphrodites were transferred to fresh 35-mm NGM plates (one worm per plate, technical quadruplicates) and allowed to lay eggs for 24 h. Then, adults were removed, and plates were kept for an additional 24 h to allow egg hatching. For each hermaphrodite, the total number of offspring during the first day of adulthood and the percentage of unhatched eggs were scored using a stereomicroscope MZFLIII (Leica, Wetzlar, Germany).

### 2.4. Germline Apoptosis

For germline apoptosis assay, *ced-1p*::GFP (MD701) worms were used. CED-1 is a transmembrane receptor that mediates apoptotic cell corpse engulfment in the nematode. Thus, using fluorescently labelled CED-1 allows the identification and scoring of gonadal sheath cells that surround each apoptotic corpse [34,35,36]. Synchronized L4 worms were exposed to CEES or HN2 in M9 for 30 min. Afterwards, worms were placed on NGM plates and incubated at RT for 24 h. Adult hermaphrodites were manually picked and immobilized on microscopic slides with 2% agarose pads and 5 mM NaN_3_. The number of apoptotic germ cells (GFP-positive) observed in the death zone loop was scored in one of two gonadal arms in at least 25 worms per sample using an Axiovert 200M (Carl Zeiss AG, Oberkochen, Germany) fluorescence microscope (40× magnification). Average values for each sample were taken for statistical analysis. Importantly, in all experiments involving manual evaluation (germline apoptosis, survival, and reproduction assays), samples were blinded.

### 2.5. Neurotoxicity

To evaluate the potential neurotoxic effect of mustards, transgenic worms BY200 (*dat-1p*::GFP)—which are frequently applied in early screening of neurotoxicants (Ijomone et al., 2021)—were used. DAT-1 is a sodium-dependent dopamine (DA) transporter which is expressed in eight DAergic neurons in *C. elegans*. Typically, this and similar strains are inspected under a fluorescence microscope to detect and manually score morphological features of degeneration, such as dendritic puncta and also shrunken or missing soma [37,38,39]. Here, we applied an automated approach inspired by previous studies on similar strains demonstrating the utility of fluorescence-activated worm sorting to detect alterations of dopaminergic neurons based on fluorescent signal reduction [40]. Synchronized L4 BY200 worms were exposed to CEES or HN2 in M9 for 30 min. Then, worms were placed on NGM plates and incubated at 20 °C for 24 h. Next, worms (A1 hermaphrodites and their offspring) were collected from plates into 15 mL tubes and washed three times with M9. The washing step allowed a substantial reduction of larvae content, and as a result mostly A1 were present in the sample. Such samples were transferred to 50 mL tubes with 20 mL of M9 and analysed using a Copas FlowPilot (Union Biometrica, Holliston, MA, USA) worm sorter. For data evaluation, the FlowJo^TM^ v.10.9.0 software (BD Biosciences, Franklin Lakes, NJ, USA) and the following strategy were applied: First, adult worms were selected based on their size (axial length) (time of flight; TOF) and optical density (extinction; EXT) (Figure S1a). Next, the mean fluorescence signal was obtained from the selected population (Figure S1b) and the values were normalized to the negative control (M9). Approximately 1000 worms were analysed per sample. Additionally, photos of representative samples were taken with the confocal microscope Zeiss LSM 880 Airyscan (Carl Zeiss AG, Oberkochen, Germany).

### 2.6. NAD^+^ Levels

For NAD^+^ analysis, worms at various life stages were treated with CEES or HN2 in M9 for 30 min. One sample consisted of approximately 10,000 L4 or 1500 adult worms. NAD^+^ extraction was performed immediately (0 h) or at later time points, after CEES and HN2 exposure. For that purpose, worms were placed on NGM plates and collected 2 h, 4 h, or 24 h later. To extract NAD^+^, worms were resuspended in 0.5 mL ice-cold M9, and placed on ice. Subsequently, 24 µL of 3.5 M perchloric acid was added, and samples were incubated for 15 min on ice. Next, samples were subjected to three cycles of freezing (in liquid nitrogen) and thawing, followed by sonication (3 × 5 s on ice, power 10%) with Sonopuls HD2070 (Bandelin, Berlin, Germany). Next, samples were centrifuged (1500× *g*, 10 min, 4 °C). Following this, 350 µL of 0.33 M K_2_HPO_4_ (pH 7.5) was added to the supernatant, which was incubated for 15 min on ice, snap-frozen in liquid nitrogen, and stored at −80 °C until NAD^+^ analysis. The pellet was stored at −20 °C until total protein analysis. For NAD^+^ enzymatic cycling assay [41], samples were thawed and centrifuged (1500× *g*, 10 min, 4 °C), and 40 µL (in technical triplicates) was transferred into a microplate. Subsequently, 160 µL of buffer (0.5 M NaOH, 0.25 M H_3_PO_4_,) and 100 µL of the reaction mixture (0.34 M bicine-NaOH pH 8.0, 2.9 mg/mL BSA, 1.4 mM MTT, 14.3 mM EDTA, 1.7 M EtOH, 5.7 mM phenazine ethosulfate, 0.14 mg/mL ADH) were added to each well. The reaction mixture was also added to wells with 200 µL of NAD^+^ standard (Sigma–Aldrich, St. Louis, MO, USA) prepared freshly in the buffer (0.5 M NaOH, 0.25 M H_3_PO_4_,) in a concentration range of 0.05–0.5 µM. The plate was incubated for 30 min at 30 °C, and absorption was measured at 550 nm with 690 nm as a reference wavelength in an Infinite M200 Pro microplate reader (Tecan, Männedorf, Switzerland). The NAD^+^ concentrations were calculated from a standard curve and normalized to the protein level. The total protein analysis was performed on sample pellets dissolved in 500 μL 0.1 M NaOH using a Pierce^TM^ BCA Protein Assay Kit (Thermo Fischer Scientific, Waltham, MA, USA) according to the manufacturer’s protocol.

### 2.7. Statistical Analysis

The experiments in this study were performed in biological replicate numbers as indicated in figure legends. Data were analysed with GraphPad Prism v.9.2.0 software (GraphPad Software, Boston, MA, USA). The used statistical tests were indicated in figure legends.

## 3. Results

**Figure 1:**
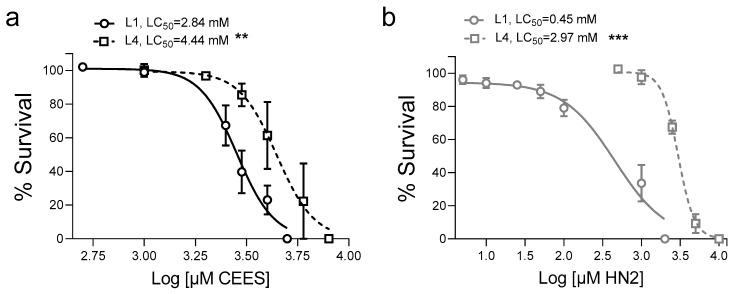
Lethality of *C. elegans* larvae L1 and L4 after CEES or HN2 exposure (**a**,**b**). Synchronized WT (N2) worms were exposed to alkylating agents for 30 min in M9. Next, approximately 30 worms were transferred onto 35 mm NGM plates in technical triplicates; 24 h later, dead and alive worms were scored, and the percentages of alive worms were normalized to solvent control (0 mM). Results were expressed as mean ± SEM (*n* = 3 biological replicates). For statistical analysis, two-way ANOVA was performed; ** *p* < 0.01, *** *p* < 0.001 vs. L1.

**Figure 2:**
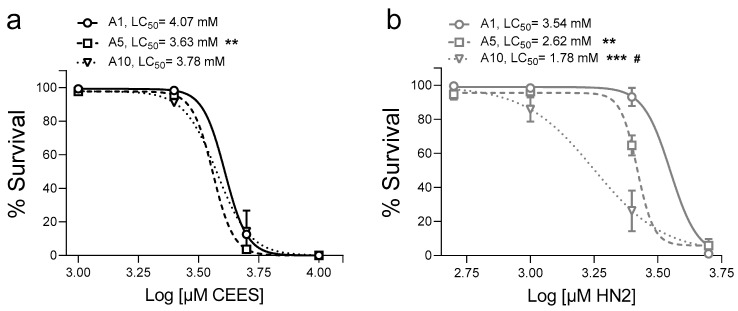
Lethality of adult *C. elegans* after CEES or HN2 exposure (**a**,**b**). Synchronized WT (N2) worms were cultivated on NGM + FUdR until adulthood day 1 (A1), day 5 (A5), or day 10 (A10), when worms were collected from plates and exposed to alkylating agents for 30 min in M9. Next, approximately 30 worms were transferred onto 35 mm NGM + FUdR plates in technical triplicate and incubated at 20 °C; 24 h later, dead and alive worms were scored, and the percentages of alive worms were normalized to solvent control (0 mM). Results were expressed as mean ± SEM (*n* = 3 biological replicates). For statistical analysis, two-way ANOVA was performed; ** *p* < 0.01, *** *p* < 0.001 vs. A1, # *p* < 0.05 vs. A5.

Toxicity of CEES and HN2 in *C. elegans* exhibited compound, life cycle, and age specificities. In general, HN2 was more toxic than CEES, with LC_50_ values in the high µM or low mM range (Figure 1 and Figure 2), which is in line with data from other models [42,43]. Importantly, the effects were dependent on the worms’ developmental stage: L1 exhibited the highest susceptibility, whereas for L4, LC_50_ increased 1.6-fold for CEES (Figure 1a) and 6.6-fold for HN2 (Figure 1b). Previous studies showed that worms’ survival was unaffected by HN2 concentrations up to 180 µM for L1 and 270 µM for L4 [44]. When compared to L4, young adult worms (adulthood day 1, A1) showed slightly increased sensitivity towards CEES (Figure 2a) with LC_50_ reduced from 4.44 mM to 4.07 mM, but decreased sensitivity towards HN2 (Figure 2b), with LC_50_ increased from 2.97 mM to 3.54 mM, although the values cannot be directly compared, as FUdR was only used in the culture of adult worms (Figure 2). The susceptibility increased further with advanced age (adulthood day 5 and 10: A5, A10), especially for HN2 (Figure 2b), for which a dramatic decline in survival was evident for A10 worms. Notably, for every life cycle stage, there was no difference in the survival of worms exposed to M9 or 1% solvent (0 mM).

**Figure 3:**
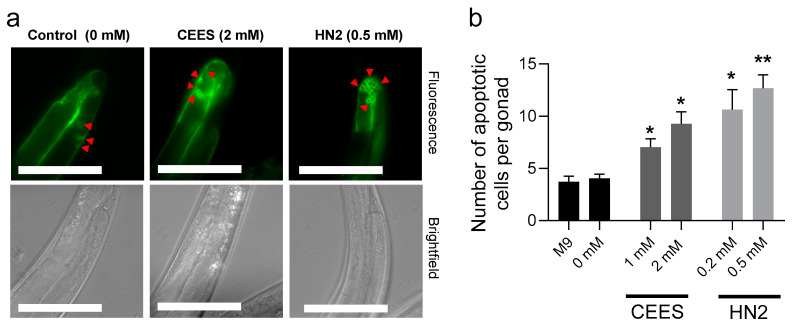
Germline apoptosis after CEES or HN2 exposure. Synchronized *ced-1p*::GFP (MD701) worms at the L4 stage were exposed to CEES or HN2 for 30 min in M9. Next, worms were placed on NGM plates and incubated at RT; 24 h later, adult hermaphrodites (at least 25 per sample) were manually picked and examined under a fluorescence microscope. The number of apoptotic germ cells (GFP-positive) observed in the gonad death zone loop (red triangles) was scored, and means for each sample were calculated. The scale bar represents 100 µm (**a**). Results were expressed as mean ± SEM ((**b**), *n* = 3 biological replicates). For statistical analysis, unpaired two-tailed *t*-tests were performed; * *p* < 0.05, ** *p* < 0.01 vs. solvent control (0 mM).

**Figure 4:**
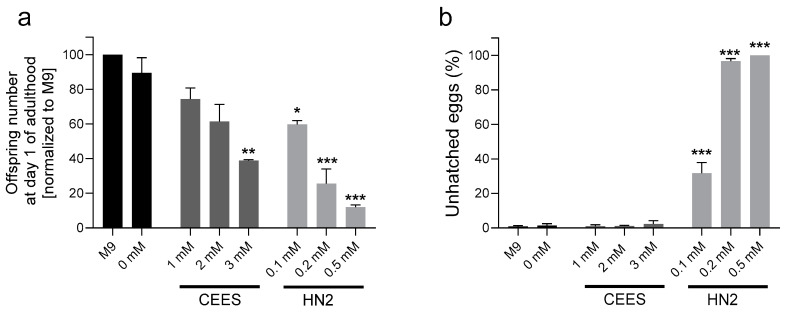
*C. elegans* offspring number and embryonic lethality after CEES or HN2 exposure. Synchronized WT (N2) L4 worms were exposed to CEES or HN2 for 30 min in M9. Next, worms were placed on NGM plates and incubated at 20 °C; 24 h later, adult hermaphrodites were manually picked and placed individually (technical quadruplicates) on 35 mm NGM plates and incubated at 20 °C for 24 h. For each hermaphrodite, the number of offspring during the first 24 h of adulthood was scored. Means for each sample were normalized to control (M9) ((**a**), *n* = 3 biological replicates). Additionally, the number of eggs unhatched during the following 24 h was scored and normalized to the offspring number ((**b**), *n* = 3 biological replicates). Results were expressed as mean ± SEM. For statistical analysis, a one-way ANOVA with Dunnett’s multiple comparisons test was performed; * *p* < 0.05, ** *p* < 0.01, *** *p* < 0.001 vs. solvent control (0 mM).

A stronger HN2 effect was also observed for germline apoptosis (Figure 3a): low-toxicity concentrations in young adult worms significantly increased the number of apoptotic cells in their gonads, up to twofold for CEES and threefold for HN2 (Figure 3b). The higher toxicity of HN2 to germline cells was also shown in the reproduction assay (Figure 4). Both CEES and HN2 lowered reproduction, but the effect was much more pronounced for HN2: 0.1 mM HN2 already significantly reduced offspring number during the first day of adulthood (by 33%), which was further enhanced by increasing HN2 concentrations (Figure 4a). Moreover, a significant fraction of embryos released from HN2-treated mothers did not hatch (Figure 4b), indicating high embryonic toxicity. The CEES effect was less pronounced, and a concentration of 3 mM CEES was needed to significantly reduce (by 56%) egg number (Figure 4a), and those eggs were viable (Figure 4b). Notably, the offspring number for M9 normalization samples was 102 ±13 and was not significantly different from the solvent control (“0 mM”).

**Figure 5:**
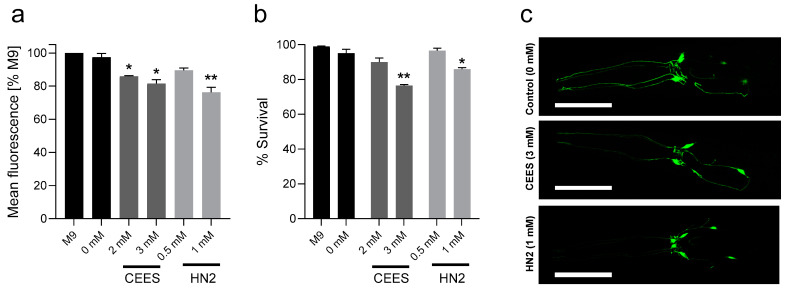
*C. elegans* dopaminergic neurons toxicity after CEES or HN2 exposure. Synchronized *dat-1p*::GFP (BY200) L4 worms were exposed to CEES or HN2 for 30 min in M9. Next, worms were placed on NGM plates and incubated at 20 °C for 24 h. Adult (A1) worms were collected and analysed using a worm sorter for the decrease in fluorescence intensity ((**a**), *n* = 4 biological replicates). The survival of BY200 was also determined: after treatments, worms were transferred onto 35 mm NGM plates in technical triplicates; 24 h later, dead and alive worms were scored, and the percentages of alive worms were calculated for each sample ((**b**), *n* = 3 biological replicates). Representative photos of dopaminergic neurons in the head area were taken with the confocal microscope; the scale bar represents 50 µm (**c**). Data are expressed as mean ± SEM. For statistical analysis, unpaired two-tailed *t*-tests were performed; * *p* < 0.05, ** *p* < 0.01 vs. solvent control (0 mM).

A potential neurotoxic effect of alkylating agents has been investigated using the BY200 strain expressing GFP-tagged DA transporter DAT-1. Both mustards induced a dose-dependent decrease in the mean GFP fluorescence (Figure 5b). For 2 mM CEES, the fluoresce was reduced to 85%, whereas for 3 mM CEES to 82% (Figure 5a). These concentrations correspond to decreased worm survival to 90% and 77%, respectively (Figure 5b). For 0.5 mM HN2, the mean fluoresce was reduced to 90%, and for 1 mM HN2, it was reduced to 76% (Figure 5a), while survival was 97% and 86%, respectively (Figure 5b). These data suggest a stronger neurotoxic effect of HN2 compared to CEES, although one must be wary with interpretation, as at the current state, we cannot completely exclude the possible impact of dead worms on the outcome. However, the dead worms were much smaller (undeveloped larvae L4) than surviving adults A1 and were removed with high efficiency from the sample during the washing process before the analysis (undocumented observation).

**Figure 6:**
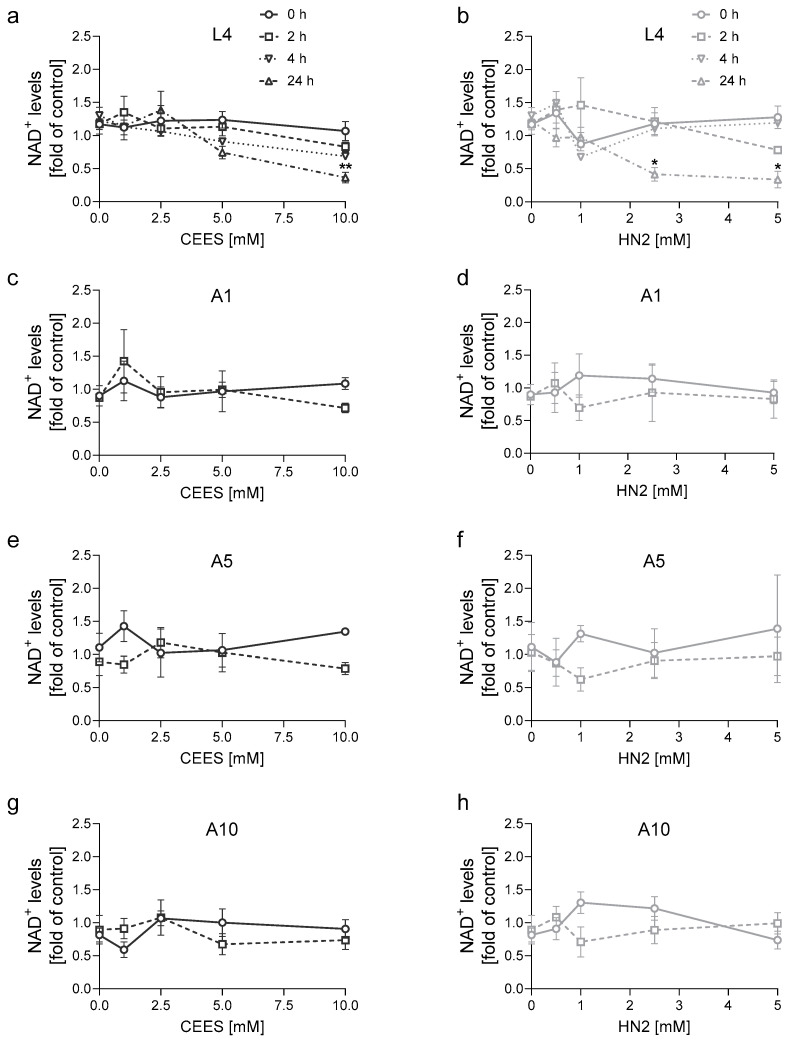
NAD^+^ levels in *C. elegans* after CEES or HN2 exposure. Synchronized WT (N2) worms at different life cycle stages: larvae L4 ((**a**,**b**); *n* = 1–3 biological replicates), adulthood day 1 (A1; (**c**,**d**); *n* = 2–3 biological replicates), day 5 (A5; (**e**,**f**); *n* = 2–3 biological replicates), or day 10 (A10; (**g**,**h**); *n* = 2–3 biological replicates) were exposed to CEES or HN2 for 30 min in M9. NAD^+^ was extracted from whole worms immediately (0 h) or after incubation for 2 h, 4 h, or 24 h at 20 °C on NGM + FUdR plates. NAD^+^ levels were measured with a cycling assay and normalised to the total protein levels measured with a BCA assay. Results were normalised to the value of the control sample (M9) and expressed as mean ± SEM. For statistical analysis, two-way ANOVA with Dunnett’s multiple comparisons test was performed, * *p* < 0.05, ** *p* < 0.01, vs. solvent control (0 mM).

As lower NAD^+^ levels have been detected in mustard toxicity in vertebrate cells [22,23,24,25], we asked whether a similar phenomenon can be observed in the nematode. The total NAD^+^ levels in whole-worm extracts were measured immediately (0 h) as well as 2 h, 4 h, and 24 h after mustard exposure (Figure 6). We did not observe any changes in NAD^+^ levels during early time points following mustards exposure, although a decrease was observed after 24 h (investigated in L4 worms only) (Figure 6a,b). Again, HN2 exhibited a greater effect, as 2.5 mM HN2 already significantly reduced (by 66%) NAD^+^ levels (Figure 6b). Notably, this concentration is lower than LC_50_ for L4 worms (Figure 1b). CEES led to a significant NAD^+^, lowering only at a concentration of 10 mM (Figure 6a), which is predicted to be 100% lethal for L4 worms (Figure 1a). For adult worms, NAD^+^ levels were analysed either immediately (0 h) or 2 h after CEES and HN2 exposure, as we were particularly interested in early toxic events. CEES had an overall low impact on the cofactor change at any worm age (Figure 6c,e,g). For HN2, a trend (not significant) towards reduction at 2 h was observed, especially for 1 mM HN2 (Figure 6d,f,h). However, the effect was not observed for higher concentrations. Notably, the absolute NAD^+^ levels were not significantly different between L4 and A1, although older worms seemed to have higher NAD^+^ content (Figure S2a). The embryos in the A1 sample might contribute to these high NAD^+^ levels, as the egg sample had the highest NAD^+^ content among the stages analysed (Figure S2a), and when adult worms were incubated with FUdR (Figure S2b) (as in the experiment shown in Figure 6), the NAD^+^ content in A1 was substantially lower. Interestingly, A5 and A10 showed increased NAD^+^ contents by 44% and 52%, respectively, compared to A1 (Figure S2b).

**Figure 7:**
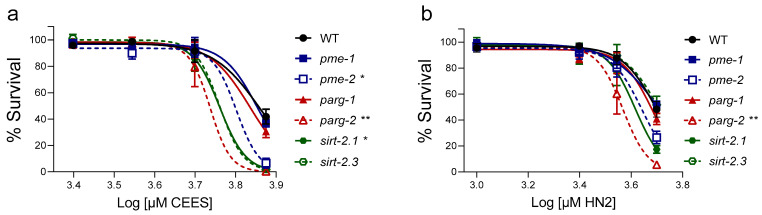
Lethality of *C. elegans* with deficient NAD^+^-dependent system after CEES or HN2 exposure (**a**,**b**). Synchronized WT (N2) and mutant worms: *pme-1*(*ok988*) (RB1042), *pme-2*(*ok344*) (VC1171), *parg-1*(*gk120*) (VC130), *parg-2*(*ok980*) (VC641), *sirt-2.1*(*ok434*) (VC199), *sirt-2.3*(*ok444*) (RB654) were cultivated on NGM until adulthood day 1 (A1), when worms were exposed to alkylating agents for 30 min in M9. Next, approximately 30 worms were transferred onto 35 mm NGM plates in technical triplicates and incubated at 20 °C; 24 h later, dead and alive adult worms were scored, and the percentages of alive worms were normalized to solvent control (0 mM). Results were expressed as mean ± SEM (*n* = 3 biological replicates). For statistical analysis, two-way ANOVA was performed; * *p* < 0.05, ** *p* < 0.01 vs. WT.

In order to examine if NAD^+^-dependent enzymes involved in genotoxic and stress response contribute to the worms’ response to CEES and HN2 treatment, several mutant strains lacking genes related to NAD^+^-dependent pathways involved particularly in DNA damage repair were investigated, i.e., ARTDs (*pme-1* and *pme-2*), poly (ADP-ribose) glycohydrolases (*parg-1* and *parg-2*) and sirtuins (*sirt-2.1* and *sirt-2.3*) (Figure 7). The *pme-2*, *parg-2* and *sirt-2.1* young adult (A1) worms showed increased susceptibility to CEES with LC_50_ reduced by 12%, 24%, and 20%, respectively, compared to WT. The *sirt-2.3* LC_50_ was also reduced by around 20% (not significant) (Figure 7a). The *parg-2* mutant animals were also significantly more susceptible to HN2, whereas *pme-2* and *sirt-2.1* showed a similar but not statistically significant trend, with LC_50_ reduced by 26%, 12%, and 17%, respectively (Figure 7b).

## 4. Discussion

In this study, we investigated the use of the multi-cellular model organism *C. elegans* to study molecular mechanisms associated with the toxicity of alkylating agents, i.e., the sulphur mustard analogues CEES and HN2. We demonstrated that in nematodes, the two surrogates of SM show effects similar to those observed in other models. Nitrogen mustard was more toxic than CEES at each stage of *C. elegans* development (Figure 1) and aging (Figure 2), confirming its higher toxicity previously observed in other systems. For example, in rats, the difference between HN2 and CEES in LD_50_ was 25-fold [43], whereas in human keratinocytes HaCaT, it was 16.4-fold [42]. The relatively low toxicity difference between CEES and HN2 when compared with other systems might be due to the treatment protocol (brief, 30 min exposure) or differences in toxicokinetics [45]. Apart from effects on general survival, HN2 also showed particularly high toxicity to proliferating germline cells, manifested in elevated germ cell apoptosis (Figure 3) as well as reduced offspring number (Figure 4a) and survival (Figure 4b). In other studies in *C. elegans*, 16 h of exposure with 80 µM HN2 resulted in a slight, nonsignificant reduction of progeny survival, accentuated in DNA repair mutants [46], and 200 µM HN2 led to a slight (approx. 20%) but nonsignificant decrease in egg hatching following 19 h exposure to young adult worms [47]. The stronger effect for 200 µM HN2 in our study (Figure 4b) might be due to differences in experimental design. Notably, toxicity to the germline was observed for concentrations much lower than to the parent organism: 1 mM CEES (Figure 3) or 0.1 mM HN2 (Figure 4) already affected the germline, whereas in the maternal population (L4), the toxic effect was observed for 3 mM CEES or 2.5 mM HN2, respectively (Figure 1). This demonstrates that investigating germline allows a sensitive evaluation of (geno)toxicity.

In the present study, we also showed a reduced expression of DA transporter DAT-1 (Figure 5a–c), suggesting the neurotoxic effects of SM analogues in the nematode. DA is a neurotransmitter whose deficit is well known for its role in movement disorders, and the development of neurodegenerative diseases such as Parkinson’s disease (PD) [48]. DAT regulates DA neurotransmission by transporting extracellular DA into the intracellular space. Thus, the absence or reduction of DAT function results in impaired synaptic DA homeostasis [48]. Previous studies showed SM neurotoxicity in various experimental models as well as cognitive and emotional disorders in veterans exposed to this compound, as recently reviewed in [49]. Neurological complications after SM poisoning encompassed sensory nerve impairments [50], motor nerve disturbances [51], retinal abnormalities [52], chronic neuropathic symptoms [53], and mental health problems such as anxiety, depression, or cognitive decline [54] and persisted even decades after exposure [55]. Neurotoxicity in vitro has been demonstrated upon SM [56] and HN2 exposure [57], and multiple evidences exist from in vivo studies. For example, chronic SM exposure resulted in neurobehavioral impairments, oxidative stress, and apoptosis in the brains of male Swiss Albino mice [58]. HN2 exposure led to neuronal degeneration [59] accompanied by oxidative stress and lipid peroxidation in the rat brain [60]. The antioxidant response was impaired in the brains of rats exposed to SM [61], and multiple stress markers were observed in the brains of hairless mice after CEES topical exposure [62]. Moreover, intratracheal CEES infusion to guinea pigs resulted in neuroinflammation, α-synuclein accumulation, and decreased DA transporter expression [63], which is in line with our observations. Further studies are needed to link the reduced DAT-1 expression with the other neurotoxic and neurological changes observed upon mustard exposure.

The nematode showed an age-specific response to mustards, with L1 worms being the most susceptible (Figure 1 and Figure 2). This might result from different toxicokinetics due to different cuticle properties and/or differential expression of genes involved in ADME [45]. With further development to L4 worms, resistance first increases but then decreases with advanced age. This is particularly the case for HN2 (Figure 2b). The reason behind increased susceptibility in advanced age is highly interesting, yet the underlying molecular mechanism needs to be clarified in future studies. Candidate mechanisms might be cuticle damage or other aging-induced physiological or molecular changes [64,65,66,67]. For instance, age-dependent depletion of cellular NAD^+^ has been linked to disease in mammals [19], and other groups have shown a progressive decrease in NAD^+^ levels during the aging of the nematode [66,68]. However, in this study, we did not observe any difference in NAD^+^ levels between A5 and A10, although both groups exhibited slightly higher NAD^+^ levels than A1 (Figure S2). There was also no significant difference between NAD^+^ levels in L1, L4, and A1 worms (Figure S2), although the NAD^+^ content seemed to increase with larval development, which might partially explain the increasing resistance to the genotoxicants at those stages (Figure 1 and Figure 2). The differences between CEES and HN2 in age-dependent susceptibility are indicative of toxicodynamic differences, considering the similar physico-chemical properties of the two compounds. Thus, compound-specific effects suggest age-dependent dysfunction of the complex repair process of HN2-induced DNA crosslinks, although this has yet to be determined. Importantly, the DNA repair capacity has been shown to correlate with *C. elegans* longevity [69,70]. Alternatively, HN2-induced ICLs may have a stronger toxic effect due to their negative impact on gene transcription, since transcriptional elongation speed has been shown to increase with age [71].

In previous studies, mustard exposure led to a partial depletion of NAD^+^ levels ex vivo [21], in vivo [22,23], or in vitro [24,25]. In *C. elegans*, mustards did not cause a substantial change in NAD^+^ levels shortly after CEES and HN2 exposure (Figure 6), although significant NAD^+^ depletion has been observed at later time points (24 h), particularly after exposure to HN2 (Figure 6b): L4 worms showed a dose-dependent effect, which was significant for 2.5 mM and 5 mM HN2 (Figure 6b), concentrations which correspond to 67% and 9% survival (Figure 1b), respectively. CEES treatment led to significant NAD^+^ depletion observed 24 h after exposure (Figure 6a) but only at the lethal concentration of 10 mM CEES (Figure 1a), indicating that it was likely a consequence of the nematode death. For adult and aging worms, NAD^+^ levels were slightly affected, but without a clear, dose-dependent pattern when early time points (0 h and 2 h post-treatment) were analysed (Figure 6c–h). Together, these results suggest that in *C. elegans*, massive NAD^+^ depletion does not occur as an early event in mustard toxicity. Although several studies showed NAD^+^ depletion following mustard exposure [21,22,23,24,25], others did not report such pronounced changes [42,72], indicating that this effect is protocol- or system-specific. Nevertheless, the functional role of NAD^+^-dependent systems in the response to mustard exposure has been confirmed by the increased susceptibility of worms lacking genes coding homologues of human ARTD (*pme-2*), PARG (*parg-2*), and SIRT (*sirt-2.1*) (Figure 7), strongly suggesting that in specific cellular or subcellular microenvironments, NAD^+^ indeed plays a significant role in toxic responses, even though this did not affect global whole-worm NAD^+^ levels. In this experiment, once again, the effect was compound-specific, with significantly higher susceptibility to CEES for *pme-2*, *parg-2*, and *sirt-2.1* mutants (Figure 7a), whereas upon HN2 treatment, only *parg-2* survival was significantly reduced, while *pme-2* and *sirt-2.1* showed a nonsignificant trend (Figure 7b). The higher susceptibility of those mutants indicates the protective role of respective molecules during mustard exposure, but the detailed mechanisms are yet to be established. For example, a detailed analysis of the activation of those molecules following alkylating agent exposure would help to clarify their role in the nematode. The results are in line with previous in vitro study, where pharmacological inhibition of ARTDs increased the number of micronuclei formed after CEES and sensitised HaCaT cells to CEES and SM but not to HN2 exposure [42]. Other DNA damaging factors and stressors have been linked to changes in NAD^+^-dependent pathways in the nematode: Worms exposed to high doses of ionizing radiation displayed a rapid drop in NAD^+^ content and increased accumulation of PAR [73]. Moreover, PME inhibition resulted in reduced progeny survival rate after exposure to ionizing radiation, likely due to impaired DNA repair response [73]. The *pme-1* worms showed increased sensitivity to cisplatin (alkylating agent forming intra- and interstrand crosslinks) treatment, reflected in reduced brood size and viability [74]. Knockdown (RNAi) [75] or knockout [76] of both PARG-1 and PARG-2 genes resulted in enhanced sensitivity of worms to ionizing radiation. PARGs in the nematode have been shown to coordinate DNA damage repair [76,77,78] and to promote axon regeneration, whereas ARTD activity had a suppressing effect [79]. In mammalian cells, SIRTs utilise NAD^+^ in their catalytic activity, which is predominantly protein deacetylation. This process regulates multiple molecular pathways, including transcription and DNA repair [80] or response to stress [81]. Overexpression of SIRT-2.1, the ortholog of mammalian nuclear SIRT1, has been shown to extend *C. elegans* lifespan [82] and to protect axons from mutant polyglutamine-induced dystrophy [83]. Interestingly, the knock-out of *sirt-2.3*, which is the ortholog of mammalian mitochondrial SIRT4, has been shown to protect nematode neurons from degeneration [84].

Limitations of the study: According to the CGC data, the deletion mutant strains were not outcrossed to the parental strains, except *pme-2*, which was outcrossed 7×. This means that unwanted background mutations might be present in those strains and influence the response to the stressors. Moreover, the effect of reduced DAT-1 expression, although significant, must be interpreted with caution, as we could not completely exclude the presence of a small number of dead worms in the analysed samples. Additionally, the functional effects of reduced protein expressions have not been investigated. These limitations must be addressed in future studies.

## 5. Conclusions

This study demonstrates that exposure to alkylating agents, the SM analogues CEES and HN2, results in compound-specific and life-cycle- and age-dependent susceptibilities of nematodes. Our data indicate a higher (geno)toxic effect of HN2, particularly in germline cells. Moreover, our data reveal toxicity to the DA system as well as the protective role of NAD^+^-dependent systems in the response to stressors. Collectively, this study provides evidence that mustard exposure results in a similar response in *C. elegans* as in the other, more traditional models, such as rodents and cell lines. Therefore, the nematode provides an attractive, 3R-compliant alternative to those models and can be applied in further mechanistic studies deciphering mustard toxicity or examining therapeutic interventions, including effects at different life cycle stages as well as organismic age.

## Data Availability

All relevant data are included in the main manuscript or the Supplementary Material file. Raw data are available upon reasonable request from the corresponding authors.

## Supplementary Materials

The following supporting information can be downloaded at: https://www.mdpi.com/article/10.3390/cells12232728/s1, Figure S1: The FlowJo^TM^ analysis of fluorescence signal in BY200 (*dat-1p*::GFP) worms following exposure to alkylating agents; Figure S2: NAD^+^ levels in *C. elegans* at different life cycle stages.
