# Supplementary material for: Life-Cycle-Dependent Toxicities of Mono- and Bifunctional Alkylating Agents in the 3R-Compliant Model Organism C. elegans
Source: Cells. 2023 Nov 29;12(23):2728. doi: 10.3390/cells12232728 (PMC10705807; doi:10.3390/cells12232728)
Supplement: Supplementary file 1 [file cells-12-02728-s001.zip › cells-2673132-supplementary.pdf]

Supplementary Information

Life-Cycle-Dependent Toxicities of Mono- and Bifunctional Alkylating Agents in the 3R-Compliant Model Organism *C. elegans*

Joanna Ruszkiewicz 1,*, Lisa Endig 1, Ebru Güver 1, Alexander Bürkle 1 and Aswin Mangerich 1,2,*

1 Molecular Toxicology Group, Department of Biology, University of Konstanz, 78457 Konstanz, Germany;

endiglisa@web.de (L.E.); ebruguver96@gmail.com (E.G.); alexander.buerkle@uni-konstanz.de (A.B.)
2 Nutritional Toxicology, Institute Nutritional Science, University of Potsdam, 14469 Potsdam, Germany
* Correspondence: joanna.ruszkiewicz@uni-konstanz.de (J.R.); mangerich@uni-potsdam.de (A.M.)

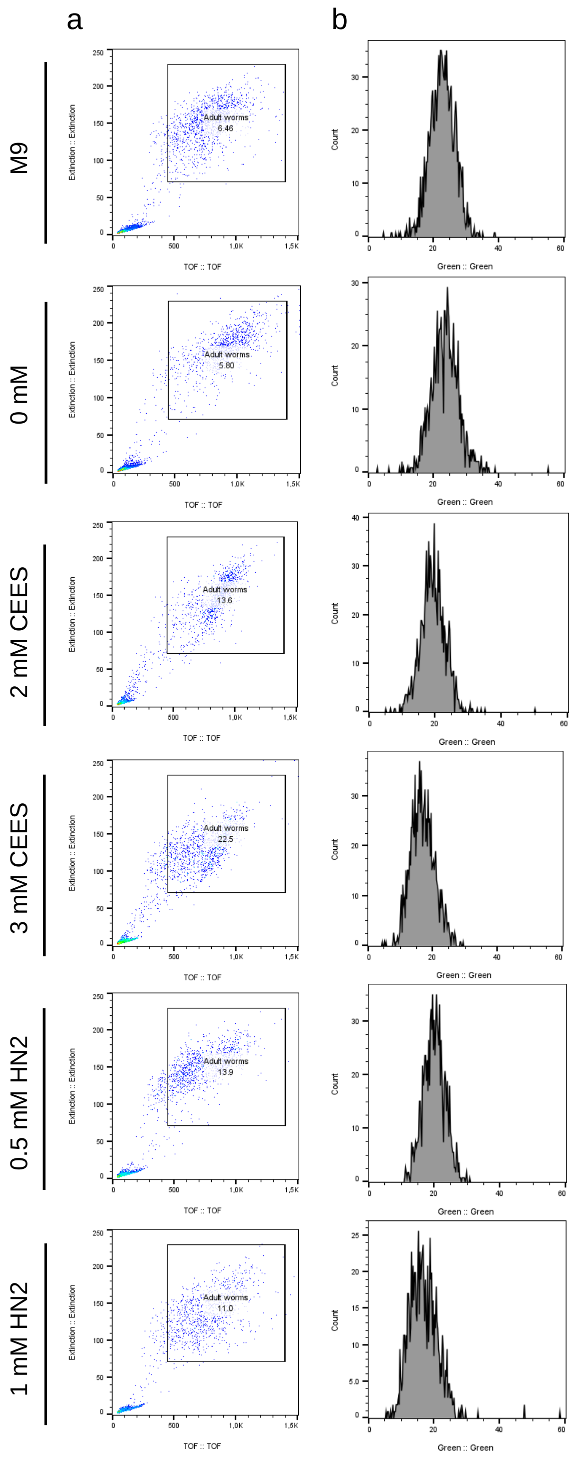

Figure S1: The FlowJo™ analysis of fluorescence signal in BY200 (*dat-1p::*GFP) worms following exposure to alkylating agents. (a) Adult worms were selected based on their size (time of flight; TOF) and optical density (extinction; EXT). (b) For the selected population the mean green fluorescence signal was acquired.

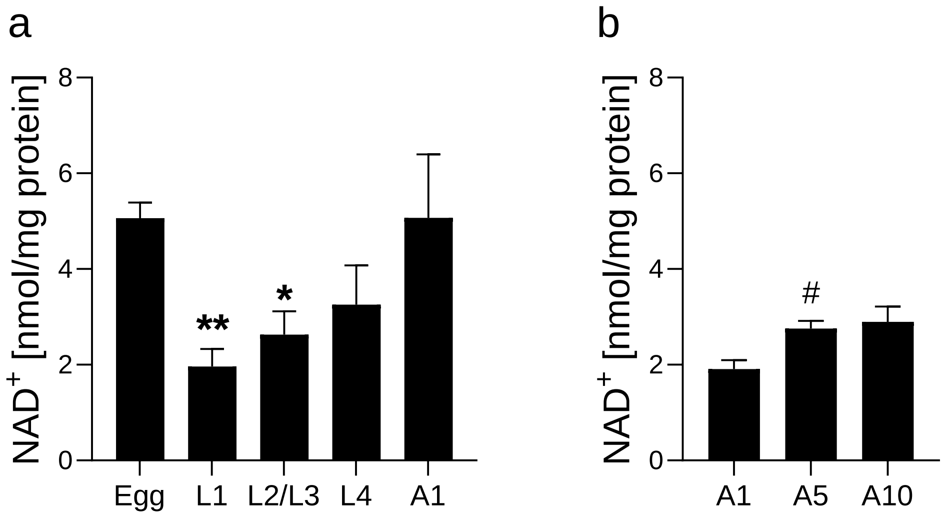

Figure S2: NAD$^+$ levels in *C. elegans* at different life cycle stages. (a) NAD$^+$ was extracted from eggs (n=3 biological replicates) or synchronised WT (N2) worms grown on NGM plates until the life cycle stages: L1 (n=6 biological replicates), L2/L3 (n=8 biological replicates), L4 (n=9 biological replicates), A1 (n=7 biological replicates). (b) NAD$^+$ was extracted from WT (N2) worms grown on NGM + FUdR plates from L4 until respective days of adulthood: day 1 (A1), day 5 (A5), or day 10 (A10) (n=3 biological replicates). NAD$^+$ was measured with cycling assay and normalised to the total protein levels measured with BCA assay. Results were expressed as mean ±SEM. For statistical analysis unpaired two-tailed t-tests were performed; *p<0.05, ** p<0.01 vs Egg; # p<0.05 vs A1.
